# Strategic approaches to improve equine breeding and stud farm outcomes

**DOI:** 10.14202/vetworld.2025.311-328

**Published:** 2025-02-13

**Authors:** Imdad Ullah Khan, Aswin Rafif Khairullah, Asfand Yar Khan, Atta Ur Rehman, Imam Mustofa

**Affiliations:** 1 Department of Clinical Sciences, Faculty of Veterinary and Animal Sciences, Gomal University, Dera Ismail Khan, Khyber Pakhtunkhwa, Pakistan; 2 Research Center for Veterinary Science, National Research and Innovation Agency (BRIN), Jl. Raya Bogor Km. 46 Cibinong, Bogor 16911, West Java, Indonesia; 3 Department of Clinical Sciences, Faculty of Veterinary and Animal Sciences, Gomal University, DI Khan, KPK, Pakistan; 4 Division of Veterinary Reproduction, Faculty of Veterinary Medicine, Universitas Airlangga, Jl. Dr. Ir. H. Soekarno, Kampus C Mulyorejo, Surabaya 60115, East Java, Indonesia

**Keywords:** economic empowerment, equine breeding management, fertility enhancement, hormonal therapy, reproductive efficiency, seasonal breeding

## Abstract

This review explores advanced strategies for enhancing fertility and optimizing reproductive outcomes in equine breeding programs. Horses, being seasonal breeders, present unique reproductive challenges influenced by environmental and physiological factors such as photoperiods, hormone cycles, and aging. Key approaches discussed include hormonal therapies, artificial light manipulation, and nutritional supplementation to improve ovulation and conception rates during the breeding season. Specific hormones such as gonadotropin-releasing hormone analogs, equine follicle-stimulating hormone, and progesterone are analyzed for their roles in synchronizing estrus and increasing ovarian activity. The document also emphasizes the significance of dietary strategies, particularly the inclusion of omega-3 fatty acids, L-arginine, and essential vitamins, in improving reproductive health. In addition, the review underscores the importance of stallion management, addressing factors such as testicular health, age, and environmental stress. Practical methods to mitigate seasonal infertility and improve foaling rates through better reproductive management of mares and stallions are detailed. These insights aim to assist stud farm owners in maximizing breeding efficiency and achieving higher economic returns. The primary goal of this review is to provide a comprehensive guide to practical interventions that increase the productivity and sustainability of equine breeding operations.

## INTRODUCTION

Equines are essential for supporting lower-middle-class and poor communities working in areas such as agriculture, construction, transportation, and tourism, often under tough weather conditions. Improving reproductive efficiency directly benefits these communities by ensuring a steady supply of healthy working animals, which helps sustain their livelihoods [1]. Another aspect of the significance of the equine species is its popularity as a pet, race, and for business purposes. Desirable offspring and bloodlines are selected for breeding to produce high-quality offspring that can be sold for a large profit. Selective breeding of equines is becoming increasingly popular in several nations as a profitable means of selling offspring [2].

Thoroughbred mares have become highly sought-after in the racing industry because they are bred with top-quality stallions to produce foals with enhanced racing skills. The selection of the broodmare is determined by lineage, racing performance, physical structure, and, occasionally, disposition [3]. A number of racehorse owners prefer their horses to foal early in the year, but this can be difficult because of the short breeding season and varied gestation periods [4]. Breeders use various methods to ensure foals are born as early as possible, ideally on January 1. In the Northern Hemisphere, they prefer foals to arrive in January, while in the Southern Hemisphere, August is the preferred month.

Young Thoroughbreds are typically classified based on their age when they are put up for sale: foals (newborns), yearlings (1-year olds), and 2-year olds. Early-born foals are housed indoors, which provides them with nutritional benefits and reduces the risk of bone fractures. In addition, their increased interaction with humans has a positive impact on their behavior [5]. According to breeders, foals born earlier are more likely to fetch a higher price at auction because they are also anticipated to perform better than foals born later [6]. To enhance reproductive efficiency in mares, it is important to consider different environmental factors, such as age, season, and feeding, as well as pathological factors such as reproductive diseases and parasitic infestations. These considerations are necessary to prevent economic losses caused by the high expenses associated with maintaining brood mares throughout the year with low fertility and conception rates [7].

Mares typically ovulate 24–48 h before the end of estrus, but their ovulation timing is less predictable than that of other animals, such as ewes and cattle. Accurately determining a mare’s reproductive condition is challenging, often leading to lost reproductive cycles and lower pregnancy rates during the breeding season. To improve mare fertility and conception rates during the breeding season or to obtain foals earlier in the year, the estrus cycle is selectively manipulated using exogenous hormones [8], artificial light treatment [9], and selection of a suitable breeding period [10]. Other key aspects to consider for a successful equine breeding program include stallion well-being, environmental stress, and food supplementation. This review aimed to explore and analyze strategic approaches and interventions to enhance reproductive efficiency in equine breeding programs, with a focus on improving fertility, optimizing breeding practices, and addressing environmental and physiological challenges.

## REPRODUCTIVE MANAGEMENT OF MARES

Mare is a seasonal long-day breeder. Mares bred in different hemispheres exhibit different reproductive behaviors. Mares raised in the northern hemisphere have a reproductive season that consists of anestrus in the winter, a spring transition (about mid-February to mid-March), the ovulatory season (April-September), and a fall transition back to anestrus [11]. The ovarian activity begins at the end of January in the northern hemisphere, but follicular development is sluggish and does not reach 21 mm in diameter (mean diameter 16 mm). This is followed by a rapid increase in follicular activity, with follicular diameters exceeding 21 mm over the period of 8 days, beginning 54 days before ovulation. This follicular development continues until the start of the ovulatory/breeding season in March and April [12].

### Selection of breeding season

Selecting the optimal breeding season is important for improving the breeding potential of mares. When the reproductive cycle coincides with optimal environmental conditions, this can help breeders improve the conception rate of mares. As the seasons change and daylight increases, mares transition from anestrus to their active reproductive cycle. This shift plays a crucial role in follicle development and overall reproductive efficiency. At the beginning of the breeding season, longer daylight boosts mares’ fertility, growing larger follicles (≈30 mm). By the end of the breeding season, activity declines, and follicles reduce in size [13–15]. The temperature and humidity index had no effect on the pregnancy rate throughout the breeding season, but the success rate of embryo transfer was found to be higher during the early season, i.e., the spring transition, than during the late autumn transition [10]. The estrus period is long during March and April. However, as summer temperatures and day length increase, especially in July, the estrus period shortens, indicating a negative impact of increased daylight, i.e., 15 h, 40 min) and high ambient temperature, i.e., 21.4 ± 0.52°C [16]. A similar pattern of decreased estrus duration with the progression of the breeding season was observed in Arab purebred mares maintained in Northern Tunisia [17]. It has been found that short estrus cycles/estrus periods have lower conception rates than normal estrus cycles/periods [18].

Seasonal changes affect the size and function of the corpora lutea and uterine edema. Ultrasound examination shows that the corpora lutea formed in late summer are smaller and less vascularized than those formed during the early or mid-reproductive seasons [19, 20]. In addition, endometrial edema scores are lower in late autumn [21]. In autumn, the corpora lutea may produce insufficient progesterone, thereby making pregnancy more difficult [22]. Supplementation with exogenous progesterone can prevent pregnancy loss in autumn-bred mares [23]. The breeding season also affects the duration of gestation and fetus development. A study by Akourki *et al*. [24] found that mares that gave birth during winter had a longer gestation time than those that gave birth during summer. Nevertheless, season could exert an adverse influence on fetus development. According to Beythien *et al*. [25], the metabolic rate of mares decreases during winter, which can potentially delay the growth and size of the developing fetus during later stages of pregnancy.

**Figure 1:**
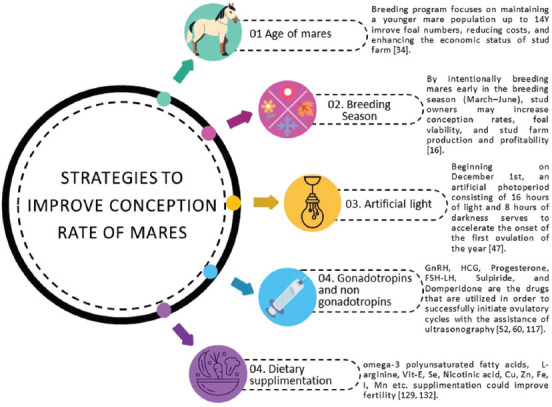
Comprehensive approaches to improve mare conception rates [https://www.canva.com/pro/].

Thus, stud owners can increase conception rates, improve foal viability, and raise the general productivity and profitability of their stud farm business by deliberately breeding mares early in the breeding season. In addition, it will help avoid potential fetal losses during gestation in mares bred during autumn (Figure 1).

### Age of the mares

The age of a mare can affect its reproductive function due to a decrease in the amount of pre-ovulatory follicles, resulting in a reduced pregnancy rate per estrus cycle [26] in mares older than 19 years compared with young mares aged between 2 and 4 years. According to Claes *et al*. [27], older female horses experience slower growth of their ovarian follicles, longer periods of time between ovulations, smaller follicle size, and decreased numbers of mature follicles. Small-sized follicles exhibit decreased expression of angiogenic, anti-apoptotic, and proliferative receptors such as epidermal growth factor receptor, Ki-67, vascular endothelial growth factor, luteinizing hormone receptor, and B-cell lymphoma 2 [28]. In addition, they show altered expression of growth differentiation factor and bone morphogenic protein-15 [29] and disrupted transforming growth factor β-signaling pathway [30]. These molecular changes not only hinder the growth of developing follicles but also diminish the viability and lifespan of the released ovum.

As the mares get older, the number of antral follicles decreases while the concentration of progesterone in the blood increases [31]. The processes of extracting follicles and recovering oocytes decreased with increasing age of the mare [11]. The number of oocyte recoveries was greater in mares aged 6–10 and 11–15 years than in those aged >16 years [32].

The rate of early embryonic loss increases with age [33]. Metaphase 2 oocytes in 16-year-old mares exhibit higher chromosomal misalignment, variable spindle length, DNA dispersion, and thicker metaphase plates than those in 14-year-old mares [34]. The embryos of 12-year-old mares had lower mitochondrial (mt) DNA than those of young mares, which may contribute to early embryonic loss [35]. Increased maternal age had no effect on embryo recovery rate, diameter, or total RNA content, but genes expressing mitochondria, protein metabolism, mitotic signaling, and adhesion pathways were negatively affected, resulting in a low survival rate [36].

Mares’ aging is connected with physical degradation of the uterine wall, which reduces conception rates and increases early embryonic death. Endometrial biopsies of older mares reveal extensive eosinophilic and inflammatory cell infiltration as well as larger intrauterine fluid collection. On gestation day 15, old mares exhibit increased uterine contractility, which may be associated with early embryonic loss [37]. Subclinical endometritis is more common in older mares (16–23 years old), with increased secretion of arachidonic acid metabolites such as prostaglandin E2, prostaglandin-1α, and leukotriene-C4, which deteriorate the immunoendocrine balance [38] and reduce the expression of progesterone receptors [39], resulting in significantly reduced fertility. Another factor is diminished vascular supply to the reproductive tract, which is linked to endometrial degradation and early embryonic death [40].

Androgens synthesized by the adrenal glands of female horses play a role in the functioning of the ovaries and the maturation of follicles. The androgen concentration is influenced by advancing age. Mares older than 16 years exhibit reduced levels of testosterone, dehydroepiandrosterone, and androstenedione compared to mares younger than 13 years [41]. Dehydroepiandrosterone and androstenedione both stimulate the development of enzymes, such as aromatase (P450arom, P450ss), which act as precursors for the synthesis of estradiol in the ovarian granulosa cells of mares [12].

Thus, with a strategic breeding program that focuses on maintaining a younger mare population (up to 14 years old), stud owners can potentially improve foal numbers, reduce costs, and enhance the overall economic viability of stud farm operations (Figure 1).

### Duration of light or photoperiod

Horses are seasonal breeders; mares’ yearly breeding season and circannual rhythm of mares are governed by photoperiods in conjunction with melatonin production [42]. Mares in the Northern Hemisphere enter anestrus during the winter and resume their reproductive cycle in the spring [43]. Environmental factors influence ovarian activity; for example, as spring transitions begin, increasing day durations increase the start of the breeding cycle, allowing foals to be born and reared in higher temperatures and in more nutritious pastures [44]. The length of the daylight rises as summer begins. Increased photoperiod has an inverse influence on melatonin generation through pineal gland stimulation. Melatonin inhibits gonadotropin-releasing hormone (GnRH) from the hypothalamus, which reduces follicle-stimulating hormone (FSH) and luteinizing hormone (LH) production in the anterior pituitary gland. When the pineal gland’s melatonin synthesis is diminished due to prolonged daylight, GnRH levels rise, followed by LH and FSH. Thus, the gonadotropin surge initiates the estrus cycle in mares following the shift from winter anestrus [45, 46].

Experiments have shown that increasing the length of light during winter transition using an artificial source can boost early seasonal ovarian activity. Artificial light treatment 2 months before foaling in winter helps to avoid early seasonal (March-April) ovarian inactivity and could be a useful strategy to boost conception rates in mares and obtain foals in the early months of the year [47]. In the northern hemisphere, foals born in January or February outperform those born later, showing better stamina, immunity, and higher annual earnings [6].

Experiments showed that starting December 1^st^, extending daylight to 16 h, and keeping darkness to 8 h can speed up the first ovulation of the year. This can be achieved using a 100–150 W light bulb in a 3.6 × 3.6 m stall to mimic longer days [48]. After artificial lighting, it takes about 60–90 days for mares to ovulate, reflecting the long summer days. In other words, mares usually begin their cycles in February or March [47].

The light spectrum responsible for manipulating the circannual rhythm is shortwave blue light [49]. Various light sources, such as incandescent, fluorescent, and direct sunlight, have varied spectral compositions, whereas sunlight contains a high concentration of shortwave blue light. Photoperiod modification using artificial blue light during the anestrus period produces the year’s early estrus cycle [50]. A head-worn mask that emits low-level blue light into one eye is both cost-efficient and successful in advancing the equine mating season [49]. Blue light (50 lux light-emitting diode [LED] light, 468 nm) directed at one eye may help mares ovulate earlier. In addition to ovulation in mares, it affects the gestational age and the developing fetus. In mid-December, blue light is administered by head-worn light masks from 08:00 to 23:00 every day [51]. The efficiency of artificial blue light is increased when combined with direct sunlight during the day [44].

This manipulation of the mare’s natural photoperiod is a common practice in equine breeding to advance the breeding season and produce foals earlier in the year. By providing the mares with additional light exposure (150–200 watt bulb or 50 lux blue LED) during the winter months (1^st^ December), breeders can take advantage of the mare’s physiological response and breed them sooner, resulting in foals being born earlier in the year. Photoperiod management allows equine breeders the ability to synchronize foal production with market demands, prioritizing the health and well-being of their animals (Figure 1).

### Exogenous regulation of follicular growth by gonadotropins

#### Equine-FSH (eFSH)/recombinant eFSH (reFSH) and equine LH (eLH)/recombinant LH (reLH) ratios

Equine pituitary extract (EPE), which contains eFSH and eLH, promotes ovulation in seasonally anestrous mares [51]. In a favorable reproductive status, it appears that the majority of anestrous mares successfully ovulate following EPE therapy, with around 50% having ≥2 ovulations. The efficiency of EPE increases with follicle size. Mares in deep anestrus with follicles larger than 25 mm in diameter responded better to EPE compared to those with follicles smaller than 15 mm [52]. In a similar study, transitional mares with small follicles (20–25 mm in diameter) had a lower ovulation rate (29%) and longer interval to ovulation than transitional mares with larger follicles (30–35 mm), all of whom ovulated in a much shorter interval with EPE treatment [10].

EPE products are not commercially accessible; therefore, they are not used in practice. However, a purified equine-FSH product (eFSH; Bioniche Animal Health, Bogart, GA) with an FSH: LH ratio of 10:1 may be commercially offered. A daily injection of 12.5 mg eFSH to transitional mares increased follicular growth and accelerated the first ovulation of the year [53]. Different EPE dosages (0.50 mg or 0.85 mg) were examined in mares receiving progesterone and estradiol therapy. Treatment responses in terms of follicular growth and ovulation were similar between the two doses tested [54].

reFSH is equally efficient as eFSH in stimulating the development of numerous follicles to a size of 35 mm or greater [55]. reFSH has been found to stimulate the development of follicles in cyclic mares, as reported by Jennings *et al*. [54] and Meyers-Brown *et al*. [56]. A study conducted in 2013 found that reFSH treatment followed by human chorionic gonadotropin (hCG) administration was highly effective in inducing ovulation in deep anestrous mares, with an ovulation rate of 76.7% [57]. As mentioned earlier, eFSH showed greater efficacy in mares going through cycle and transition phases. However, mares in profound anestrus (with a follicle size ranging from 10 to 16 mm in diameter) did not response well to treatment [57]. The use of reFSH alone or in conjunction with reLH while maintaining the natural photoperiod was found to be successful in stimulating follicular development and inducing ovulation in mares experiencing deep anestrus. reFSH was administered alone or in combination with 0.65 mg IM twice daily with an 8-h interval until the follicle size reached 32 mm. When the follicle size reached 35 mm after 36 h, hCG (2500 IU) was injected intravenously (IV) to induce ovulation [52]. Previous research has shown that reFSH alone is more effective than when combined with reLH in promoting follicular development and the synthesis of insulin-like growth factor 1 and estradiol within the follicles [58].

eFSH or reFSH administration can help induce and synchronize follicular development, leading to improved ovulation rates and increased chances of successful breeding and conception. This treatment is particularly beneficial for mares who are experiencing reproductive challenges, such as delayed puberty, anestrus, or irregular estrous cycles. Incorporating the strategic use of eFSH or reFSH into an equine breeding program can improve overall reproductive efficiency and sustainability.

#### GnRH, GnRH analogs, and hCG

GnRH or its analogs stimulate the anterior pituitary gland to release LH and FSH, which trigger ovulation in seasonally anestrus mares [59, 60]. Several methods of administration have proven effective, including single or multiple injections [61], hourly pulses delivered through external or peristaltic pumps [62], and constant infusion using subcutaneously implanted osmotic mini-pumps [63]. The effectiveness of GnRH treatment depends on several factors, such as the method of administration, size of the follicle at the time of treatment, and stage of anestrus [64]. According to Papas *et al*. [65], mares in transition with follicles larger than 25 mm in diameter are more likely to respond to treatment than those with follicles smaller than 15 mm. Using 40 µg of buserelin, the ovulation rate was 100% in heavy draft mares with follicles measuring approximately 45 mm [66]. Deslorelin acetate effectively stimulated ovulation in 90% of mares during both early and late fall.

In addition, endometrial edema scores during estrus were lower in late fall than in early fall [21]. Pulsatile GnRH systems, which mimic the natural release of GnRH, may be more effective for triggering ovulation and activating the pituitary gland in mares, especially during the early and late spring transition periods [67]. However, using GnRH to induce ovulation very early, before the natural mating season, or after long-term GnRH implants may sometimes lead to issues, such as abnormal corpus luteum formation [68] and the absence of a normal estrus cycle [69]. These problems may be caused by the subsequent downregulation of GnRH receptor levels [70]. Despite this, ovulations in GnRH-treated anestrous mares are similar to spontaneous ovulations, with normal corpus luteum formation, progesterone production, and fertility outcomes [71].

GnRH or analogs administered to mares enhance LH concentrations during anestrus, estrus, and diestrus [72]. Using 100 µg of gonadorelin (a GnRH analog) resulted in an immediate increase in LH levels within 0.25 h of treatment [73]. However, when a lower dose of 40 µg of buserelin (another GnRH analog) was administered, the LH rise did not follow the same pattern [66]. Administration of 100 µg of GnRH increased blood flow to the ovaries in mares during the diestrus phase, along with elevated LH and FSH levels, compared with higher doses of 300 µg, whereas progesterone level remained unchanged regardless of the GnRH dosage [72]. Deslorelin 1.5 mg also improved the vascular supply and led to a gradual increase in LH levels [74]. These findings suggest that the GnRH dose influences the extent of LH increase. GnRH plays a key role in regulating progesterone release during the luteal phase of the estrous cycle in mares. Continuous or pulsatile GnRH infusion produces a stronger progesterone response than a single dose of a GnRH analog [72, 73].

Injecting mares with 1500 IU of hCG IV when their follicle diameter is 35 mm or larger leads to increased ovulation and conception rates. It also promotes accelerated growth of the early conceptus and raises progesterone level in the bloodstream [75]. Higher hCG doses, such as 3000 IU IV [76] or 2500 IU IV [74], result in increased follicular development and decreased endometrial folding. To save costs and prevent the development of hCG antibodies, a lower dose of 450 IU of hCG was administered IV at the Houhai acupoint, which successfully induced ovulation in donkeys [77]. When the effects of different hCG dosages (1500 IU and 3000 IU) were compared in mares, 1500 IU was more effective in inducing ovulation within 48 h, achieving the highest pregnancy rate of 93.33% [78]. The success of hCG in inducing ovulation depends on the size of the follicle, with follicles 35 mm or larger being more responsive to the hormone [10].

When comparing various GnRH analogs to hCG, no significant differences were found between buserelin, deslorelin [79], histrelin [80], lecirelin [81], or triptorelin in terms of their effects compared to hCG [82]. Deslorelin, whether in liquid form or as a pellet, was as effective as hCG in triggering ovulation [83]. The response to hCG and histrelin acetate also varied with the mare’s age. Mares under 8 years old responded equally well to either 1500 IU of hCG IV or 250 µg of histrelin acetate IM, whereas mares older than 15 years were more sensitive to histrelin in inducing ovulation during the breeding season [84].

hCG is the preferred choice of equine practitioners for inducing ovulation in mares, with a reported usage rate of 91%, compared to 64.7% for GnRH analogs. This preference is largely attributed to the lower cost of hCG in Europe [85]. Recent studies contradict earlier findings, showing that hCG induces ovulation more effectively than GnRH analogs during the transitional period, with one study reporting a 90.9% ovulation rate for hCG-treated mares compared to 37.9% for GnRH analogs [10].

Equine practitioners often recommend combining hCG and GnRH to induce ovulation because of their potential synergistic effects. However, a study found that combining a GnRH analog with hCG did not produce any additional benefits. The group treated with both GnRH and hCG ovulated at the same time as those treated with hCG, deslorelin, or histrelin [86]. In embryo-recipient mares, this combination had no impact on corpus luteum development, progesterone level, or fertility rates [87]. Changing the treatment approach may improve reproductive efficiency in subfertile aged mares. In one study, mares were given deslorelin (125 mg, i.m., every 12 h) when their follicle size reached 20 mm. Once the follicle had grown to 25 mm, hCG (500 units, i.m., every 24 h) was administered until the follicle reached 35 mm. Ovulation was triggered by treatment with 2500 IU of hCG. All mares ovulated, and the embryo recovery rate was 58.8%, which was higher than that of the non-treated group. When deslorelin was replaced with histrelin (125 µg, i.m.), the embryo recovery rate increased to 72.8% [88].

Using GnRH and hCG to stimulate ovulation in mares is an effective way to improve fertility management. These treatments allow precise control over ovulation timing, increasing breeding success. GnRH stimulates natural hormone release, whereas hCG directly triggers ovulation, helping to determine the best breeding time. This approach can improve reproductive management and veterinary care efficiency. Despite their effectiveness in stimulating ovulation, some studies have shown that hCG or GnRH analogs may not have a positive effect on overall ovarian function in mares [87].

#### Progesterone

Native progesterone and synthetic progesterone are used in anestrous mares to promote estrus and synchronize ovulation, increasing their susceptibility to breeding or embryo transfer. Regular injections of these hormones help in the transition from anestrus to a more fertile condition with increasing reproductive outcomes in mares that are not cyclic or abnormal estrus during the transitional phase in mares [10]. Some authors have claimed that the treatment does not affect the mean time to ovulation but rather serves to synchronize the onset of ovarian activity [89]. The treatment outcome depends on the stage of anestrus and ovarian activity at the initiation of treatment. Daily progesterone administration does not induce ovulation in deep anestrus, but ovulation occurs within 15 days after the end of treatment when progesterone is applied during transition [47]. The mechanism of action of progesterone during the late transitional phase is not completely understood, but it seems reasonable to assume that their administration might have a mild positive effect on FSH secretion from the pituitary gland [90].

Progesterone is supplied through various methods, including oral, parenteral, and intravaginal implants. Anestrus mares were administered oral progesterone such as allyl trenbolone (Regumate) (30 mg) [91] or altrenogest (0.044 mg/kg body weight [BW]) every day for 10–15 days. At the end of treatment estrus occurred 4.67 ± 1.83 days and ovulation happened 7.83 ± 2.82 days later [92].

When comparing the efficacy of oral altrenogest with that of intravaginal devices, both methods showed similar effectiveness in inducing ovulation [93]. Progesterone-releasing intravaginal devices (PRIDs) are commonly used to stimulate ovarian activity in anestrus mares. Although these devices can cause intravaginal inflammation, they have no adverse effects on follicle growth, ovulation, or conception rates [94]. PRIDs are inserted into the vagina for a period of 10 days. Studies on different progesterone doses loaded into these devices revealed that a dose of 1.38 g of progesterone combined with 16 h of artificial light (using 50 W reflectors) over a 10-day period effectively induced the first ovulation of the year in late transitional mares. In contrast, devices loaded with 1.9 g of progesterone did not significantly alter serum progesterone level compared with those loaded with 1.38 g of progesterone [47].

Administering HCG, GnRH, or GnRH analogs toward the end of PRID treatment improves ovulation rates. Mares were successfully ovulated when treated with HCG (1667 IU) [95] or a GnRH analog (Ovuplant, containing deslorelin acetate) [96] after achieving a follicle size of ≥30 mm in diameter following 10 or 12 days of PRID implantation. This effect may be attributed to PRID’s ability to increase the sensitivity of mature follicles to HCG [11]. Combining HCG with PRID therapy enhances ovarian function [97]. In addition, treating mares with histrelin (a GnRH analog) for 4 days at the end of a 9-day PRID treatment improved the embryo recovery rate during the breeding season [98].

PRID administration effectively induces estrus and ovulation in mares at various times of the year. However, its effectiveness is reduced during winter anestrus [99]. Administering a double PRID has been shown to enhance ovarian activity in anestrus mares that lack a corpus luteum and possess follicles measuring 25 mm or larger in diameter. This treatment induces cyclicity, synchronizes follicular waves, and stimulates ovulation [100]. A treatment regimen combining intravaginal progesterone administration through a Controlled Internal Drug Release Device-B with subcutaneous implantation of deslorelin (short-term-implant) effectively stimulated follicle growth, ovulation, and normal fertility in acyclic or anestrus mares, regardless of the season [101]. Winter anestrus mares also responded to testosterone treatment (150 µg/kg) in a manner similar to intact mares [102]. In mares experiencing lactation anestrus, administering PGF2α injections 12 days after PRID implantation successfully induced follicle development, estrus, and ovulation. Young anestrus mares exhibit a stronger response to estrus induction than older mares [92].

Parenteral treatment with a long-acting progesterone formulation BioRelease P4-LA-300 (BET Pharmacy, Lexington, Kentucky, USA) triggered ovulation in late-transition mares. A single intramuscular injection of progesterone (600 mg) at ≥25 mm follicle size on October 15^th^ promoted ovulation in 83% of mares [103]. Anestrus mares also reacted to long-acting progesterone injections. In this situation, estradiol benzoate (2.5 mg) followed by 1500 mg of long-acting progesterone generated identical uterine alterations and molecular dynamics in anovulatory mares as in cyclic mares [104].

Progesterone supplementation can effectively improve reproductive performance in transitional and cycling mares when included in a well-planned breeding strategy. However, in mares experiencing deep winter anestrus, combining progesterone with gonadotropins or estradiol may help stimulate estrus.

### Reproductive management of mares by agents other than gonadotropins

#### Dopamine antagonists and prolactin

In addition to pituitary gonadotropins, other factors influencing seasonal ovarian activity in mares include prolactin and dopamine and their effects on gonadotropin regulation. During the winter anovulatory season, dopamine concentrations in an equine cerebrospinal fluid increase [105], thereby suppressing prolactin release [106]. This suppression is mediated through dopamine’s inhibitory action at D2 receptors in the anterior pituitary, which can be blocked by dopamine antagonists [107]. The role of prolactin in initiating the reproductive cycle in anestrus mares has been confirmed in previous studies using exogenous prolactin [108]. Prolactin can directly influence the hypothalamus, as prolactin receptors located there regulate the release of GnRH [45]. In addition, prolactin receptors in the ovaries directly affect ovarian processes such as follicular development, steroidogenesis, and gonadotropin receptor expression [109]. Increased levels of ovarian steroids, such as estrogen, can further enhance this process by providing positive feedback to the hypothalamus, stimulating the secretion of GnRH [110]. Dopamine antagonist administration during the spring transition improves follicular activity in mares [111, 112]. Three dopamine antagonists – domperidone, perphenazine, and sulpiride – have been extensively studied for their effectiveness in this regard [113]. This interconnected system highlights the complex regulatory role of dopamine and prolactin in controlling seasonal ovarian activity in mares. Dopamine antagonists, such as sulpiride (200 mg/mare IM), are used to stimulate ovulation in mares. When administered daily for 36–58 days starting on February 5, ovulation occurred approximately 91 days after treatment began. This was accompanied by peak levels of prolactin, FSH, and LH in the blood [114]. Domperidone, a selective D2 dopamine receptor antagonist, can also increase prolactin levels. Multiple doses of domperidone result in significant and long-lasting (>24 h) increases in prolactin concentrations [113]. However, studies have shown that sulpiride is more effective than domperidone in deeply anestrus mares when administered daily from February 3 to February 28 [115]. The success of dopamine antagonist treatment depends on the management conditions and degree of anestrus at the start of treatment. In vernal transitional mares, ovulation occurs within 12–22 days of treatment. However, deep anestrus mares may require up to 2 months of treatment to achieve ovulation [51]. For optimal results, dopamine antagonists should be used in vernal transitional mares or the treatment should be combined with an artificial photoperiod before administering dopamine antagonists [111]. In addition, the prolactin response to sulpiride can be prolonged by delivering it in a hydrophobic carrier, such as vegetable oil or sucrose acetate isobutyrate [116].

Sulpiride’s effectiveness is significantly enhanced when combined with estradiol cypionate [116]. Pretreating seasonally anovulatory mares with estradiol increases their prolactin response to sulpiride or domperidone, leading to higher LH secretion and earlier ovulation. This approach is consistent with breeding programs targeting mid-January foaling dates [117]. Estradiol administration for 21 days during the summer can increase prolactin levels by 4–5 times in ovariectomized pony mares [118]. However, the prolactin response to sulpiride varies seasonally, peaking in June (summer) and declining to its lowest levels in December (winter) [112]. Another method to induce early ovulation involves combining sulpiride (0.5 mg/kg twice daily) with an extended indoor photoperiod [119]. Although some studies have indicated that sulpiride can stimulate follicle development and trigger ovulation in deeply anestrus mares [114, 116], other studies by Fanelli *et al*. [10], Donadeu and Ginther [12], and Panzani *et al*. [111] have reported inconsistent results. These differences highlight the need for personalized treatments and more research to improve sulpiride’s effectiveness.

In conclusion, dopamine antagonists such as sulpiride and domperidone can enhance prolactin secretion, which in turn helps hasten the onset of ovulation in transition mares, especially when combined with estradiol pretreatment.

### Dietary supplementation

Dietary supplementation is a key focus in addressing reproductive issues in animals. Maternal nutrition at conception affects gene expression and cellular function in developing offspring. Supplements often include amino acids, minerals, and fatty acids.

Supplementation with long-chain omega-3 polyunsaturated fatty acids (PUFAs) (0.06 g/kg) has been shown to improve reproductive, metabolic, and immune health in many species. PUFAs also have an immunomodulatory effect on the endometrium, enhancing early fetal conception and growth by increasing the expression of trophoblast and endoderm markers, such as GATA-binding protein-3, GATA-binding protein-4, GATA-binding protein-6, transcription factor AP2α, and ETS transcription factor-3 [120]. In addition, it promotes uterine involution when fed during the peripartum period and increases conception rates by reducing the risk of early embryonic loss [121]. In Marwari horses, supplementation with fish oil as a source of PUFA for 70 days improved follicle and corpus luteum size, as well as embryo development, 28-day post-ovulation [122]. Equine oocytes are rich in lipids, particularly PUFAs, which provide energy during oocyte maturation and early embryo development. PUFAs have a positive effect on reproductive health in healthy, non-obese mares. However, in obese mares, excess fat can lead to metabolic changes in ovarian follicles, such as increased lipid accumulation and mitochondria damage, which negatively affect fertility. This accumulation can disrupt normal follicular function and reduce oocyte quality and fertility. Supplementing diets with a specific mix of vitamins, minerals, probiotics, and antioxidants can help address these issues by normalizing lipid levels in oocytes and increasing mt activity and developmental potential [123].

When mares were supplemented with 100 g of L-arginine in their diet, ovarian blood flow improved, the size of dominant follicles, and reduced the amount of uterine fluid that accumulated after breeding [124]. L-arginine also promotes the mobilization of white adipose tissue, making it safe for older mares during gestation [125]. Supplementation with L-arginine at 0.0125% of BW led to larger fetal sizes between days 25 and 45 after ovulation in both younger and older mares compared with non-supplemented control mares, suggesting that arginine enhances embryonic growth [126]. Similarly, foals born to mares supplemented with 100 g of L-arginine during late gestation were larger in size [127].

Vitamin E and selenium supplementation, given a week before foaling or at 5-day intervals, can reduce the frequency of retained placenta, even when serum selenium concentrations are normal or below 40 ng/dL [128]. Maintaining high levels of serum Vitamin E and selenium in mares by administering 1000 mg of Vitamin E and 50 mg of selenium 3 weeks before birth has been shown to improve fertility, especially in selenium-deficient mares [129]. Early embryonic death, a major cause of pregnancy loss in mares, may be linked to nicotinamide adenine dinucleotide (NAD) deficiency. Mares fed 3 g of nicotinic acid per OS had higher NAD precursor bioavailability and improved oocyte quality [130]. In addition, micro-minerals play a key role in enhancing pregnancy rates in mares with infertility or reproductive abnormalities. Supplementing diets with micro-minerals such as selenium, copper, zinc, iron, manganese, phosphorus, and iodine during the spring breeding season has been shown to be beneficial [131].

Dietary supplementation is essential for sustainable breeding in mares and provides the nutrients needed for reproductive health. Proper nutrition improves fertility, supports estrous cycles, and helps ensure successful pregnancies, making it a key part of a successful breeding program (Figure 1).

## REPRODUCTIVE MANAGEMENT OF STALLION

Stallions are the foundation of equine breeding programs, and selection is based on pedigree, performance, conformation, and temperament. Maintaining high fertility is critical for stallions’ economic value and influence on the breeding population; therefore, mitigating variables that reduce stallion fertility should be considered.

### Seasonal impact on stallion fertility

**Figure 2:**
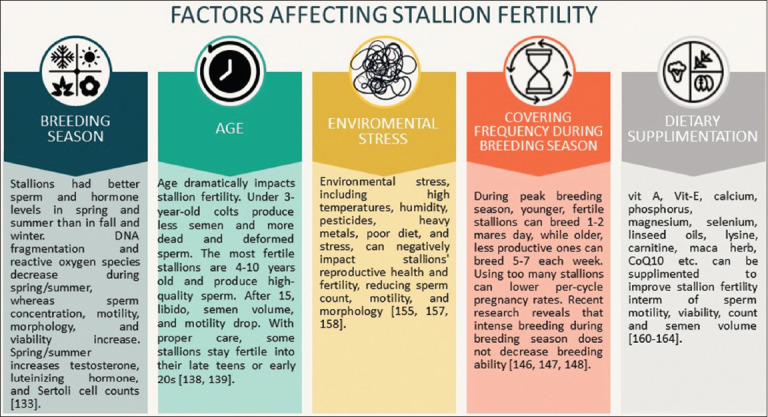
Key determinants of stallion reproductive health [https://www.canva.com/pro/].

The equine reproductive season starts in spring when stallions exhibit seasonal variations in semen quality, hormones, and sexual behavior. Sperm production and quality are mainly influenced by photoperiod changes. During the breeding season (spring and summer), stallions exhibit higher sperm concentration, better sperm motility and progressive motility, and improved sperm morphology and viability. However, sperm quality declines during the non-breeding season (fall and winter) [132]. Levels of testosterone, LH, and Sertoli cells are elevated in spring and summer, whereas sperm DNA fragmentation is lower in summer than in winter [133]. Seasonal changes significantly affect various fertility metrics, such as fertility per cycle and end-of-season fertility [134]. In winter, stallion sperm exhibits higher levels of reactive oxygen species and lipid peroxidation than in summer [135]. Kallikrein (KLKs) proteoforms in seminal plasma also contribute to temperature adaptation during reproduction. In Mangalarga Marchador stallions, increased KLK levels were associated with reduced sperm motility, larger semen volumes, and higher glucose and cholesterol levels in the seminal plasma [136]. Higher glucose and cholesterol contents in seminal plasma suggest that the reproductive system adapts to the breeding season. Glucose molecules can help stabilize the sperm membrane in anerobic conditions, such as in the uterine environment, making semen more resistant to damage (Figure 2).

### Age of the stallion

Age can have a major effect on stallion fertility, influencing various physiological and reproductive characteristics. Colts younger than 3 years produce less semen, with lower concentrations and total numbers of spermatozoa, as well as the greatest percentages of dead and malformed, non-motile spermatozoa [137]. Younger stallions (4–10 years old) tend to have the highest fertility rates. They produce high-quality sperm with sufficient motility and proper morphology. The fertility of stalks gradually decreases after they reach the age of 15 years. Older stallions may have decreased libido, semen volume, sperm concentration, and motility [138]. However, there was significant individual variance among the stallions. With proper care, some older stallions can remain fruitful into their late teens or early 20s [139]. Blood enzyme activity and lipid profiles can also affect sperm quality and cryostability in stallions of various ages [140]. Thus, understanding age-related changes in stallion fertility and semen quality is critical for breeders to make informed choices when selecting stallions for breeding operations (Figure 2).

### Testicular size and stallion fertility

Testicle size is a reliable predictor of fertility in stallions. Several studies have demonstrated a positive relationship between testicle size, sperm production, and quality. In young bulls, larger testicle sizes are correlated with higher average ejaculate volumes [141]. In a study on Arabian stallions, the scrotal width was strongly associated with the age and BW of the stallions, as well as all other testicular characteristics evaluated, including length, width, height, and total testicular volume. Scrotal breadth was also positively associated with serum testosterone levels [142]. Testicular size grows with age, with stallions 7 and older having significantly larger testes than younger stallions (2–6 years old) [143]. The number of Sertoli cells, weight, and testicular size all grow with age, stabilizing around the time the stallion reaches sexual maturity, which is roughly 4–5 years old [144].

### Frequency of ejaculation/coverage during the breeding season

A stallion can breed number of mares during a season depends on its age and reproductive health. During the peak breeding season, younger, fertile stallions (≤5 years old) may be able to breed 1–2 mares daily. Stallions older than 11 years or less fertile may only breed one mare per day or every other day. A stallion can safely breed 5–7 mares every week during the breeding season [145, 146]. Stallions can service mares more frequently throughout the breeding season; however, this higher ejaculation rate may deplete semen reserves and lower semen quality, particularly in less fertile stallions. Maintaining optimal stallion fertility requires careful monitoring of breeding frequency [147, 148]. Overuse of stallions (stallions used more than 21 times in a week) was associated with reduced per cycle pregnancy rates; hence, it should be avoided. Pregnancy loss rates were more likely to be lower in stallions that were shuttled in the previous season [149]. Therefore, compared with their older counterparts, younger stallions typically breed to more mares each day and each week throughout the mating season. Despite the findings that extreme breeding negatively affects stallion breeding efficiency and semen quality, many recent data suggest that extreme breeding during the breeding season does not affect stallion breeding capability. Stallions with larger books achieved higher rates of live foal births, according to insights from a review of 2005 breeding records conducted under consistent management conditions, which revealed that stallions with “super” and “mega” books bred an average of 3–5 mares daily during the peak breeding season (April and May), compared to those with “traditional” or “big” books, who bred only 1–2 mares/day. It seems that the point at which ejaculation frequency negatively affects fertility was not reached because stallions with a super or mega book ejaculated more often but still had the highest foaling rates [150]. Higher fertility with more frequent breeding may be explained by the natural behaviors of semiferal horses, in which stallions breed receptive mares every hour as part of their social structure. In these horses, when a stallion transitions from bachelor to harem status, his testosterone levels, libido, and sperm production increase, which enhances his fertility [151]. Similarly, studies on domesticated stallions have shown that more exposure to mares and frequent mating can improve reproductive function, suggesting that regular breeding helps maintain optimal fertility [152].

### Impact of environmental stress on stallion fertility

Environmental stress can significantly affect the fertility and reproductive health of stallions. High temperatures and humidity can disrupt sperm production and quality, with heat stress impairing testes’ ability to regulate temperature. This leads to reductions in sperm count, motility, and morphology. Prolonged exposure to heat, fever, or inflammation further intensifies the issue by increasing testicular metabolism faster than blood flow, resulting in hypoxia and disrupted spermatogenesis [153, 154].

Environmental pollutants, such as pesticides, heavy metals, and endocrine-disrupting chemicals, also pose a threat by lowering sperm counts and motility. These substances interfere with the hormonal balance and spermatogenesis, further compromising fertility [155]. In addition, poor nutrition – particularly deficiencies in essential vitamins, minerals, and antioxidants – can significantly impair sperm production and overall reproductive health [156].

Stressful experiences, such as frequent handling, transportation, and challenging social interactions, can disrupt hormonal balance and reduce libido. Chronic stress further diminishes reproductive efficiency, negatively affecting stallion fertility [157].

Addressing environmental conditions, ensuring proper nutrition, and minimizing stress are essential steps to safeguard stallion reproductive health and maximize fertility.

### Dietary supplementation

Similar to working horses, stud horses require a 10% increase in diet consisting of hay, oats, or lucerne as well as unlimited access to pasture grass. This is because stud horses have higher nutritional needs than mares or geldings (male horses who have been castrated) [158]. Additional nutrients, including Vitamins A and E, minerals (calcium, phosphorus, magnesium, and selenium), vital fatty acids (seed oils), and amino acids (lysine, carnitine), can be added to stallion feed to improve fertility [159]. Docosahexaenoic acid, in particular, is integrated into the lipid bilayer of the outer membranes of sperm, allowing for increased progressive motility and velocity as well as flexibility, compressibility, deformability, and elastic properties [160]. Supplementation with linseed oil enhances sperm vitality, osmotic tolerance, and acrosome integrity [161]. The combination and feeding of various nutrients, such as 1000 IU of Vitamin E, 2 mg of selenium as selenized yeast or 360 mg of zinc, 205 mg of selenium, and 1500 mg of Vitamin E, administered daily for 60 days improved the morphology and progressive motility of sperm in both fresh and cooled semen [162]. A combination of Vitamin E, selenium, L-carnitine, and omega-3 and omega-6 fatty acids improved sperm lifespan, motility, and acrosome integrity during *in vitro* storage, protecting them against oxidative stress [163]. Thus, the combination of specific supplements may work synergistically to enhance health and reproductive benefits.

Some nutritional supplements, like L-carnitine found in the epididymis, are believed to benefit spermatogenesis, sperm maturation, and mt activity. They may also protect germ cells from apoptosis [164]. Some thoroughbred studs use L-carnitine as an oral supplement to increase fertility. According to certain nutritional scientists, the ability of horses to produce carnitine from methionine and lysine is quite limited, making carnitine a potentially essential amino acid [159].

Vitamin E deficiency in stallions causes a more anomalous sperm with lower motility, which can be corrected with Vitamin E treatment [165]. For healthy spermatogenesis, both horses and cattle require Vitamin A and carotene because these nutrients help with sperm motility and prevent the production of aberrant sperm [166]. Dietary antioxidant Vitamins C, E, and β-carotene are correlated with sperm concentration and total progressive motility in humans, but supplementary antioxidants administered to pony stallions do not improve sperm quality to the level of a well-balanced diet [167].

Only a limited number of botanical/herbal items have been used to address and enhance equine fertility. The addition of 20 g of the herb maca (*Lepidium meyenii*) to the daily diet of stallions improved the quality of their sperm. This improvement was observed in fresh and chilled semen, with higher sperm concentration, increased motility, and enhanced resistance to sperm DNA fragmentation [168].

Co-enzyme Q10 (CoQ10) is a natural fat-soluble vitamin-like molecule found in cellular membranes and circulatory lipoproteins. CoQ10 is a strong antioxidant that exists in two forms: Fully reduced, aromatic diol form (ubiquinol) and oxidized form (ubiquinone) [169]. In stallions with low semen freezability, adding CoQ10 to the semen extender enhances post-thaw semen parameters [170]. Daily CoQ10-ubiquinol administration improves sperm quality indicators and increases plasma CoQ10 concentrations after freezing and chilling in certain stallions [171].

Thus, supplying the right nutrients through dietary supplements helps stallions maintain good reproductive health, improving fertility and sperm quality, and resulting in a successful breeding season (Figure 2).

## CONCLUSION

This review provides a comprehensive analysis of the key strategies to enhance equine reproductive efficiency, focusing on hormonal therapies, photoperiod manipulation, dietary supplementation, and environmental management. The findings emphasize the seasonal nature of equine breeding, the physiological intricacies of mares and stallions, and the significant role of targeted interventions in improving fertility rates and foaling outcomes.

The review’s primary strength lies in its synthesis of diverse strategies, offering a holistic understanding of equine reproductive management. It combines scientific insights with practical applications, bridging the gap between research and field practices. The inclusion of hormonal, nutritional, and environmental factors provides a multidimensional approach to addressing equine fertility challenges.

Despite its comprehensive scope, the review is limited by a reliance on findings from specific breeds and regions, which may not universally apply to all equine populations. In addition, some interventions discussed, such as advanced hormonal treatments, may not be feasible for all stud farms due to cost or accessibility constraints. Variability in individual responses to these interventions also warrants further investigation.

Future research should focus on breed-specific reproductive strategies and the development of cost-effective alternatives to hormonal therapies. Emerging technologies, such as precision breeding and genetic interventions, hold promise for further improving fertility outcomes. In addition, exploring the long-term impacts of dietary supplementation and artificial lighting on overall equine health could yield valuable insights. Collaborative studies across diverse geographic regions are essential to validate the applicability of these strategies on a global scale. By addressing these gaps and leveraging advancements in veterinary science, equine breeders can optimize reproductive efficiency, ultimately contributing to the sustainability and profitability of the industry.

## AUTHORS’ CONTRIBUTIONS

IUK: Collected the data and drafted the manuscript. ARK: Revised the manuscript. AYK and AUR: Data collection and image editing. IM: Critical evaluation and supervision. All authors have read and approved the final manuscript.
